# Toward a Rigorous Theoretical Description of Photocatalysis Using Realistic Models

**DOI:** 10.1021/acs.jpclett.3c00359

**Published:** 2023-04-12

**Authors:** Ángel Morales-García, Francesc Viñes, Carmen Sousa, Francesc Illas

**Affiliations:** Departament de Ciència de Materials i Química Física and Institut de Química Teòrica i Computacional de la Universitat de Barcelona (IQTCUB), c/Martí i Franquès 1-11, 08028 Barcelona, Spain

## Abstract

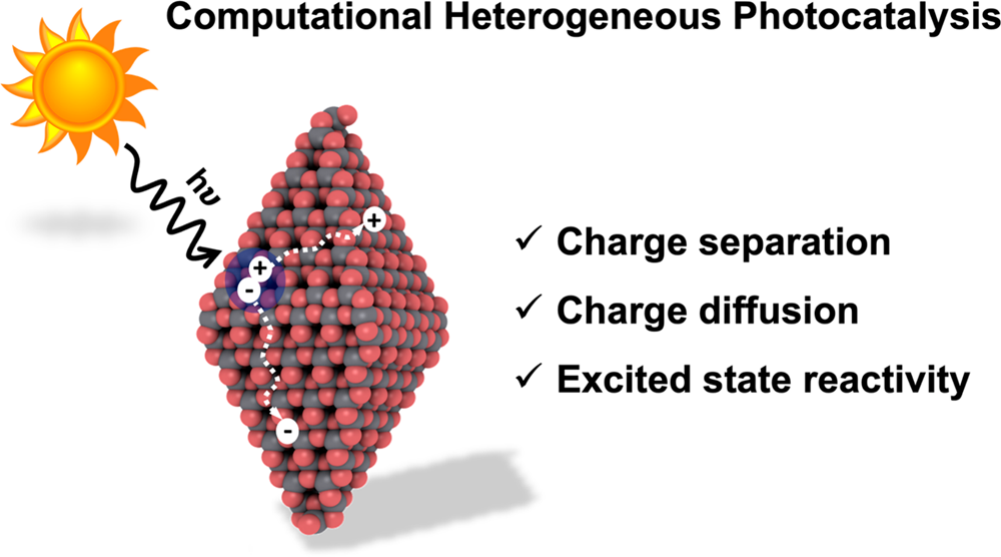

This Perspective aims at providing a road map to computational heterogeneous photocatalysis highlighting the knowledge needed to boost the design of efficient photocatalysts. A plausible computational framework is suggested focusing on static and dynamic properties of the relevant excited states as well of the involved chemistry for the reactions of interest. This road map calls for explicitly exploring the nature of the charge carriers, the excited-state potential energy surface, and its time evolution. Excited-state descriptors are introduced to locate and characterize the electrons and holes generated upon excitation. Nonadiabatic molecular dynamics simulations are proposed as a convenient tool to describe the time evolution of the photogenerated species and their propagation through the crystalline structure of photoactive material, ultimately providing information about the charge carrier lifetime. Finally, it is claimed that a detailed understanding of the mechanisms of heterogeneously photocatalyzed reactions demands the analysis of the excited-state potential energy surface.

In 1874 in his book *L’Ile Mystérieuse* Jules Verne stated “Water will be the coal of the future”.^1^ A few years later, Giacomo Ciamician, the father of modern photochemistry, suggested that fuels of the future will be produced using solar energy as the driving force.^2^ This vision envisages an elegant and ambitious pathway to convert solar energy into chemical energy, for instance through water splitting into hydrogen (H_2_) and oxygen (O_2_). This is surely one of the most important chemical processes related to energy sources,^3^ since water (H_2_O) is not only an inexhaustible natural resource but also a fully sustainable source of energy, recycled upon reaction of H_2_ and O_2_. Hydrogen thus produced becomes a renewable, sustainable, and nonpolluting fuel; in addition, solar energy becomes a renewable energy source and a major alternative to fossil fuels, possibly the best way to handle the energy and environmental crises, the two major ever-increasing challenges to our society.^4^

In 1972, almost a century after the Verne’s book release, Fujishima and Honda published a seminal short article in *Nature*(5) describing UV-light-assisted electrochemical water splitting using titania (TiO_2_) as photoanode in a photoelectrochemical (PEC) cell. This landmark paper launched research projects worldwide exploring the performance of different semiconducting materials in different applications from environmental cleaning to H_2_ production^6^ to the point of yielding nearly 85,000 articles published on the topic so far with less than 150 published before 1990. However, in spite of this tremendous scientific endeavor, no other metal oxide, nor alternative material candidate has yet been found to outperform TiO_2_ under UV light and, hence, might act as an efficient photoanode with conduction and valence band edges that straddle the redox potentials of water. In a Perspective article in this journal, Teoh et al.^7^ specified a few fundamental aspects that should be considered to succeed in the design of functional photocatalysts. These are (*i*) novel synthetic procedures to build new photoactive nanostructures; (*ii*) better understanding of the charge transport; (*iii*) the engineering of photogenerated charge delivery, so that they can reach the photoactive sites; and, finally, (*iv*) unraveling the underlying reaction mechanisms. Most, if not all, of these essential requirements persist as challenges to experimentalists and theoreticians, who should establish synergies to move forward the next generation of photoactive semiconductors.^8^

A heterogeneously photocatalyzed process starts with an out-of-equilibrium process where an electron is excited by light of appropriate wavelength/frequency. This is usually understood from a one-electron picture involving a transition from the highest occupied molecular orbital (HOMO) or valence band (VB) to the lowest unoccupied molecular orbital (LUMO) or conduction band (CB),^9^ depending on whether the photocatalysts is considered as a finite system or a periodic solid. The excitation generates a positive hole in the HOMO (or VB) and a particle in the LUMO (or CB), although higher energy levels could be involved. This is already an oversimplified, yet useful, picture since a rigorous description involves the relevant N-electron wave functions. In any case, the excited state rapidly decays to the ground state by spontaneous emission, a process in the attosecond–femtosecond time scale. Thus, the electronic relaxation competes with the formation of the exciton, a quasiparticle-like entity describing the time evolution of the hole–electron system before a spontaneous decay. Consequently, most of the created hole–electron pairs tend to recombine even if the irradiation is maintained, thus jeopardizing the photocatalyzed process.

Fortunately, in some materials, enough excitons survive upon constant irradiation that can split into charge carriers after overcoming the exciton binding energy, a quantity determined by the intrinsic electronic structure of the photoactive material.^10^ Accordingly, the charge separation process^11^ leads to the holes and electrons moving independently as influenced by their effective masses, temperature, and concentration, and they eventually can trigger the photocatalyzed redox reaction at the surface of the photocatalyst. Here one has to consider the formation of polarons, a charge carrier (electron or hole) excess localized within a potential well which is self-generated by displacing the surrounding ions.^12^ The diffusion of the charge carriers toward the surface takes place in the picosecond time scale, and this is the reason for maintaining the irradiation to supply enough hole–electron pairs, with a few being able to separate, which requires concentration gradients to move them adequately. These gradients can be produced by effective utilization of dopants, interfaces, and other surface modifications of the photoactive heterostructures.^13^ In such a case, the photocatalytic process finishes with the consumption of the charge carrier by a redox reaction on an even longer time scale. Clearly, the large asymmetries in the time scale between spontaneous emission, exciton creation, charge diffusion, and reactivity cause enhanced recombination losses.^14^ A general workflow of the heterogeneous photocatalysis process is depicted in Figure 1; this illustrates critical aspects to consider in the formulation of novel photoactive materials.

**Figure 1:**
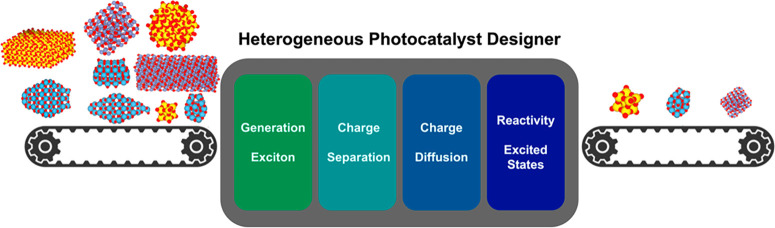
Schematic picture of the steps required to design improved photocatalytic materials: generation of the exciton in the presence of light, exciton splitting into charge carriers, and their diffusion apart. These are fundamental aspects in photocatalysis leading to the subsequent reaction in the excited state.

From a theoretical point of view, a complete study of one photocatalytic reaction requires different methods, approximations, and models as recently reported by some of us.^15^ The first aspect to consider, which many papers in the literature take as the only one, is an accurate estimation of the energy gap—usually approximated as the band gap (*vide infra*)—followed by a description of the photogenerated species, their location, character, and time evolution up to the description of the reaction mechanism in the excited state, very rarely considered.^16,17^ In fact, a very large number of studies have been reported investigating the band gap of different photoactive materials mostly considering periodic bulk and surface models of the materials of interest with just a few focusing on realistic nanostructures. Apart from the limitations of the bulk and extended surface models, most of the available computational studies report ground-state properties, such as the energy gap, estimated from one-electron energy levels rather than using more rigorously grounded techniques, such as the many-body perturbation theory (MBPT) as in *GW* methods, time-dependent DFT approaches, or configuration interaction wave functions to better approach excited-state solutions. The band theory approach has been broadly used, the case of N-doped TiO_2_ being paradigmatic^18^ but with many cases in the literature as evident from devoted reviews.^19,20^ This approach is computationally affordable for most crystalline systems and can provide valuable information regarding trends in the band gap of different materials but fails to provide results where the intrinsic nature of a photocatalytic process, involving excited states, cannot be disregarded. To reach an accurate description of the electronic properties of photoactive materials, accurate analyses of the relevant excited states are needed; this field is still in its infancy. Explicitly handling excited-state properties is expected to allow one to establish meaningful descriptors that can contribute to the design of more efficient photoactive materials.

The aim of this Perspective is to briefly review the state-of-the-art of the field indicating the commonly used approaches and, subsequently, to provide a comprehensive computational road map to describe the electronic properties and dynamics of the photogenerated species as well as ways to approach the reaction mechanisms in the appropriate excited states. We first review the computational strategies usually employed in the literature, mostly focusing on ground-state properties and suggest ways to include a proper description of the relevant excited-state chemistry.

As just mentioned, the most broadly used descriptor to ascertain whether a material is a suited photocatalyst is its band gap, usually estimated from a given density functional which is not exempt of problems.^21,22^ From the calculated band gap, one aims at finding whether it enables the absorbance of solar light in the visible spectrum region.^23^ For finite systems, such as clusters or nanoparticles, that can be easily computationally assessed by the HOMO/LUMO energy gap (*cf*. Figure 2a), which obviously depends on the chosen functional^24^ although the deviations tend to be systematic.^25^ However, under periodic boundary conditions, *e.g*., optimized bulk structures, the standard band gap approach involves the evaluation of the materials band one-electron eigenstates across the **k**-space given their band dispersion and normally tackled following certain high-symmetry paths of the Brillouin zone, taking VB and CB energy limits (*cf*. Figure 2b).^26^ Apart from the limitation from the one-electron picture, this approach neglects most of the **k**-space and can unduly yield to larger VB/CB energy gaps if such limits are not within the explored **k**-paths.

**Figure 2:**
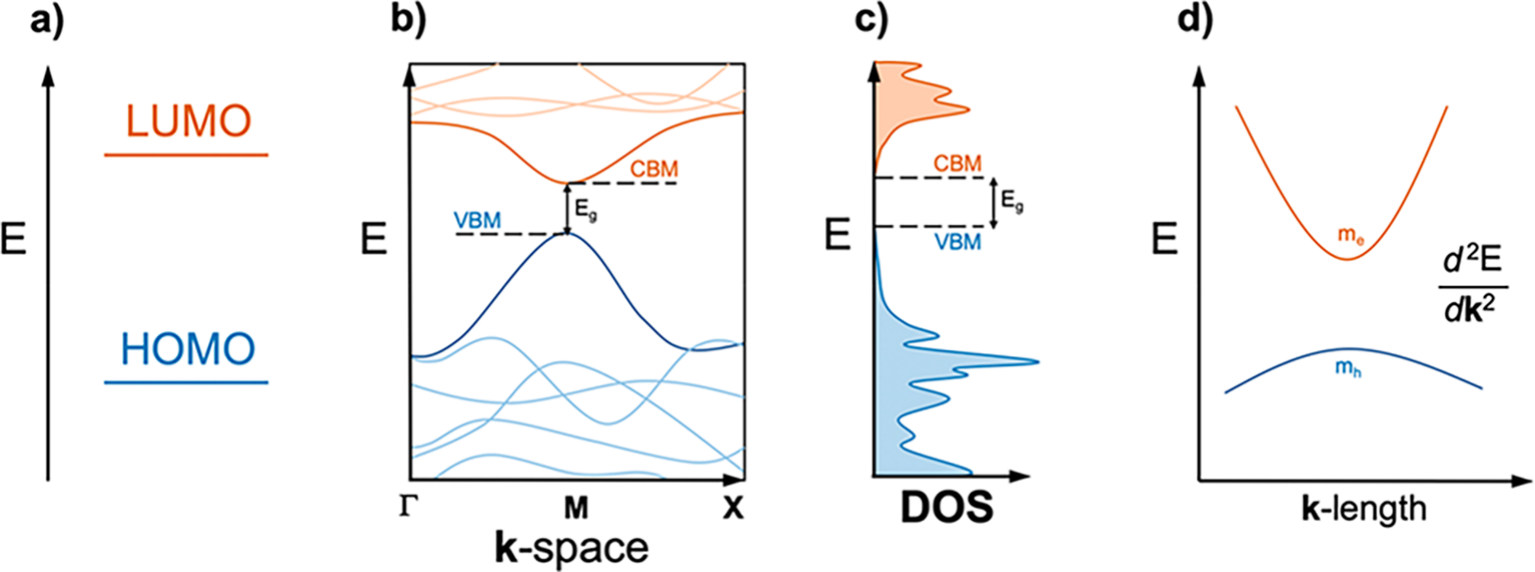
(a) Energy difference between unoccupied (orange) LUMO and occupied (blue) HOMO. (b) Electronic band structure plot along the **k**-space, including a direct band gap, *E*_g_, between the VB maximum (VBM) and CB minimum (CBM). (c) DOS displaying *E*_g_, and (d) electron (*m*_e_) and hole (*m*_h_) effective masses, inversely depending on the band energy curvature at VBM and CBM along the **k**-space.

To circumvent this issue, a complementary and more suited evaluation of the band structure energy limits is tackled by the integration across the space when plotting the density of states (DOS)^27^ (*cf*. Figure 2c), although in some cases, *e.g*., for 2D materials, a band contour can be provided, as seen, for example, in graphene and other 2D carbon-based materials.^28^ Notice that such evaluation of the band structure is also commonly approached when using surface slab models, and in all cases, they serve as a template to evaluate the band structure change when engineering the material, such as when adding dopants, surface species, or strain, to name a few. In addition, the VB and CB contours across the **k**-space can be useful to get estimates of the hole–electron charge carrier effective masses in different directions of the space (*cf*. Figure 2d) and are also used to evaluate the charge separation and avoidance of recombination.^29^

This evaluation of the band structure can be carried out at different theory levels, which can be more or less suited for such a purpose. For instance, wave function methodologies can provide accurate energy gaps, but their use on large systems may be handicapped and its implementation for periodic systems is extremely difficult although new embedding techniques are emerging which are promising.^30^ With the advent of DFT, most of the current studies rely on Kohn–Sham orbitals to carry out such an evaluation, but one has to be aware that standard DFT within, for example, the generalized gradient approximation (GGA) severely underestimates the materials band gap, a well-known consequence of the electronic self-interaction error of the Kohn–Sham implementation of DFT.^20,21^ A usual approach to counteract this is to use hybrid exchange–correlation functionals, which add a fraction of Hartree–Fock (HF) exchange energy to the DFT *ansatz*, a choice which may be also problematic,^21^ with some proposal to extract it from the material dielectric constant^31^ although this implies either experimental input or initial guess of the dielectric constant that can be refined in a self-consistent, yet nonvariational, way, although one must note that, rigorously speaking, this implies introducing a macroscopic property in the Hamiltonian which adds a semiempirical flavor to the method. In general, DFT hybrid functionals are capable of providing reliable band gap estimates, at the expense of a higher computational cost, especially when using a plane wave basis set, although one has to keep in mind that often the percentage of exchange has to be adapted to the material under inspection^20,21^ and that in range-separated functionals, such as the widely used HSE06,^32,33^ some internal parameters are far from being universal.^34^ Still, the eigenvalues gained at this level rely on a one-electron approach. More accurate and better physically grounded methods have been proposed where a quasi-particle description of electrons and holes is provided by applying Green’s functions (*G*) and screened Coulomb potential (*W*) in the MBPT based *GW* formalism,^35^ which, in any of its flavors, is capable of providing good correlations between estimated band gaps and experimental values^36,37^ although at a much higher computational cost.

The HOMO/LUMO (or VB/CB) estimates are used as well to determine the adequacy of the scrutinized materials to carry out certain photocatalytic processes, *e.g*., in the case of photocatalytic water splitting, by comparing them to the proton semi reduction potential energy, or the oxygen reduction to water, the thermodynamic limits of this process.^38^ In addition, this simple approach can be used to evaluate the doping effect,^39^ the possible electron injection or hole trapping in gap states, even the charge carrier transfer within nanoparticle facets,^28^ or in between different semiconductor materials, relevant in employed heterojunctions.^40^ Note, for instance, the discussion on the rutile and anatase TiO_2_ nanoparticles, and how the type of contact can affect the charge carrier transfers between the two polymorphs (*cf*. Figure 3).^41^ Still, such analyses have to be taken with a grain of salt, since all are based in the one-electron picture of the electronic ground state. However, no excitation is explicitly accounted for in this approach, and that, even if useful, is an uncomfortable rock-in-the-shoe since ground-state properties are alleged to be determinant in excited-state processes, which does not necessarily always have to hold true, since in the excited state one would necessarily have hole and electron charge carriers.

**Figure 3:**
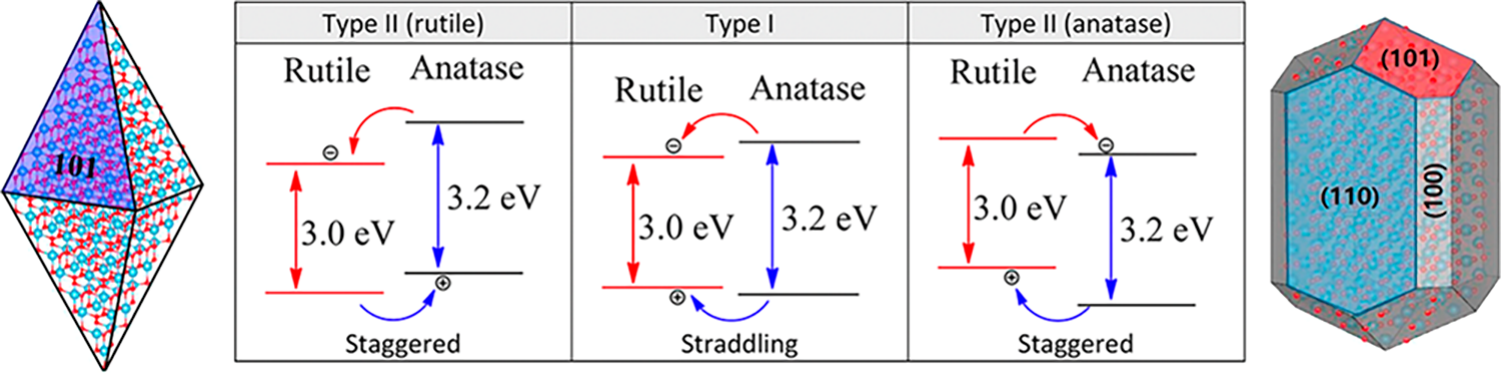
Possible TiO_2_ rutile and anatase type of contacts and level alignments, and the charge transfer they would prompt. Side images show anatase (left) and rutile (right) equilibrium Wulff shapes with the noted exposed facets. Image composed and modified from those appearing in ref (40).

Some attempts have been reported where the photogenerated holes and/or electrons are modeled in an approximated fashion. For instance, one can simply study the positively or negatively charged system by removing or adding one electron, respectively. This simple approach of estimating the electronic structure of an excited state is feasible, particularly for finite models, yet neglecting the existence of the exciton and of the hole–electron interaction. This approach can also be used in systems with periodic boundary conditions, but to avoid a Coulomb explosion due to the use of a charged unit cell, it is necessary to artificially add a counter charge density equally distributed in the system background volume. Here one should carefully analyze the supercell size to ensure convergence of the properties of interest.^42^ Another way of approaching hole and electron states separately is by adding electron traps or donors, to simulate hole or electron existence, as done by some authors by adding OH or H surface groups to slab models.^43,44^ Most often the approach still assumes that electrons get localized on surface OH groups, creating an adsorbed hydroxyl group, or that a H atom provides one electron to the system under study, becoming an adsorbed proton, which, again, does not necessarily represent the real system. A more accurate description of the dynamics of the trapped electrons in anatase surfaces has been provided by Selcuk and Selloni^45^ by means of first-principles molecular dynamics with important insight, yet dealing with the ground state of the system upon addition of electrons.

The approaches discussed so far, mostly based on the analysis of the ground state, give mainly information about the HOMO–LUMO gaps or the excitation energies of such states involved in the photocatalytic process. Except in a few cases,^29^ the study of the excited states derived from the hole–electron photogenerated exciton are not explicitly studied. As commented above, an essential step for an optimal efficiency in a photocatalytic process is the creation of excitons, which in a subsequent stage will lead to separated electrons and holes that finally diffuse to the surface where the reactions take place. Hence, a detailed analysis of the nature, degree of localization, and stability of the excitonic states is crucial for the understanding of the whole process. Despite this, only a few works explicitly tackling the excited states involved in the photocatalytic process have been published. To properly describe these excited states, the most straightforward yet simple methodology is based on time-dependent DFT (TDDFT). However, to avoid artificial electron delocalization due to the inherent self-interaction error of standard DFT, either some extent of exact exchange has to be included in the exchange functional^46^ or an on-site Hubbard *U* term has to be added.^47^ In this context, Valero et al.^48^ studied the transient absorption spectra and the character of the first singlet excited state of a series of TiO_2_ anatase and rutile derived nanoclusters by means of TDDFT calculations using hybrid functionals and a polarizable continuum model (PCM) to incorporate the effect water as solvent.^49^ Several topological descriptors were suggested to measure the degree of local/charge transfer character associated with the electronic transition. Hence, measures of the charge transfer degree, the overlap between the hole and electron charge densities, the distance between the centroids of charge of the hole and the electron (*cf*. Figure 4a), or the character of the electronic excitations from the analysis of the natural transition orbitals (NTOs) allowed the conclusion that charge separation depends on the shape and size of the nanoclusters. This permitted ranking different TiO_2_ nanoparticles in terms of their potential photocatalytic activity and as a result gives guidelines toward the rational design of TiO_2_ photocatalytic nanoparticles with suitable excited-state properties.

**Figure 4:**
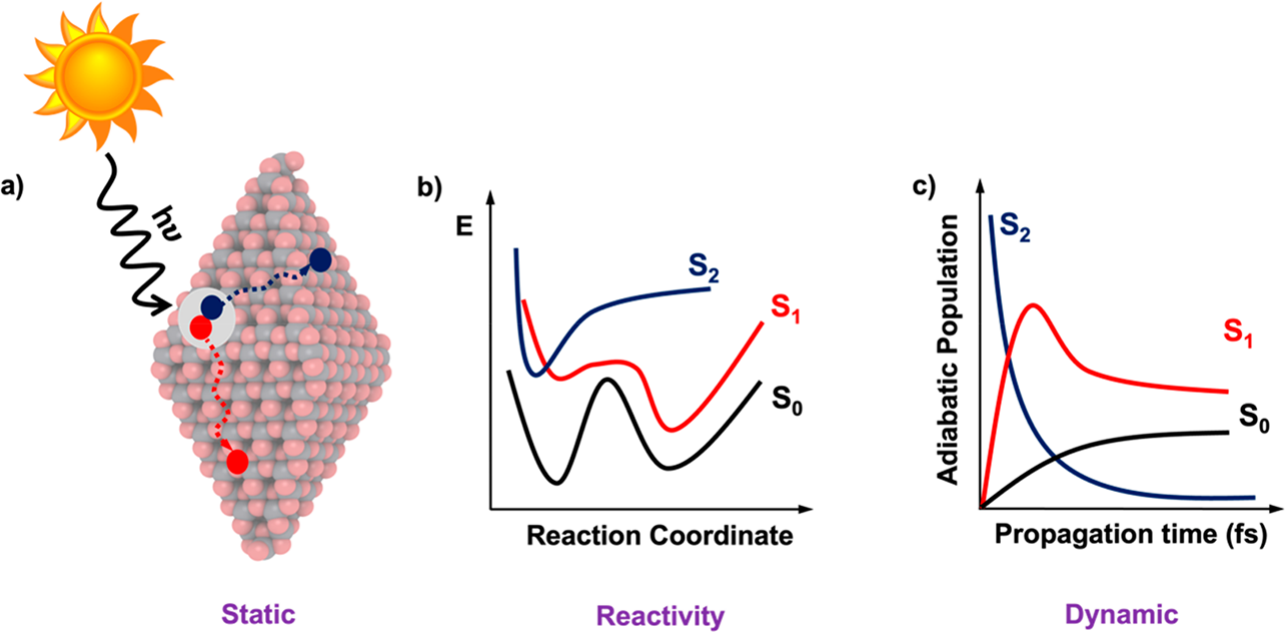
(a) Scheme of the formation exciton and generation of the electron and holes propagated through a bipyramidal TiO_2_ NP. (b) Scheme of different potential energy surface represented by ground state (S_0_) and two excited states (S_1_ and S_2_). (c) Scheme of the evolution of the population of the ground and excited states with time.

An alternative approach to tackle the singlet photoexcited state is based on open-shell DFT calculations where the two unpaired electrons are coupled to a triplet spin state instead of the spin-allowed first excited singlet. This approximation allows the structural optimization of the system in an excited-state potential energy surface which is supposed to be close to that of the singlet state (*cf*. Figure 4b). Hence, the computation of the luminescence spectra of a self-trapped exciton can be approached.^50^ The estimated error introduced by considering the triplet state instead of the singlet is expected to be small, since the energy difference between both states is mainly due to the exchange term between an electron and a hole that are located on different regions of the system. Based on this approximation, the trapping energy for the electron, hole, and exciton in the bulk, bare, and hydroxylated surfaces can be straightforwardly computed. It has been shown that for anatase TiO_2_ surfaces, the electrons and holes become self-trapped at undercoordinated Ti and O surface sites, respectively, or to isolated OH species when the surface is hydroxylated.^51^ The lattice relaxation energy related to the trapping at the surface was found to be larger than the one at the bulk, suggesting that the diffusion of the photogenerated charges from the bulk to the surface is energetically favored.

The treatment of the exciton as a triplet state not only allows one to establish the character and localization of the hole and electron at the surface but also enables the study of the photocatalytic reactions. Migani et al.^52^ described the hole–electron pair as a triplet spin multiplicity state and showed that the transfer of the hole to the photocatalyst surface takes place via a bound exciton with a strong hole–electron interaction. This approach simultaneously addresses the redox process and the hole–electron interaction, providing a better estimate of the reaction barriers, as shown in the study of the photocatalytic O–H bond dissociation of water adsorbed on the rutile TiO_2_(110) surface.^53^ Notice that this approach, at variance with the usual treatment where the hole and electron are assumed to be independent and only one of them is considered, explicitly includes the particular excitonic state although, for convenience, treats the resulting state as a triplet state. This can be justified by invoking the so-called triplet charge recombination process which occurs after photoexcitation and also impacts the global photocatalytic efficiency.^54^

Although hybrid DFT methods, once the optimal exchange–correlation functional for the particular system is tuned, can be successfully applied to study the lowest excited state of a given spin multiplicity, they exhibit limitations in the study of other low-lying excited states, charge transfer processes, or the description of strongly correlated materials, such as photoactive transition metal oxide semiconductors. As an alternative, many-electron wave function methods allow an accurate treatment of electronic exchange and correlation without dependency on the particular system and thus are a more reliable and transferable methodology to make predictions. Unfortunately, the concomitant computational cost restricts the application of these methods to systems of limited size, which, on the other side, offer a poor representation of the real system. A compromised strategy consists of using newly developed embedding techniques^29^ or splitting the system into manageable subsystems that are treated by different computational approaches. Typically, the local region of chemical interest is treated with high-level electron correlated wave functions while the crystal environment is modeled by means of lower-level embedding schemes, most often at the DFT level.^55,56^ This approach requires the determination of an embedding potential, which replaces the interaction between subsystems at the DFT level. Importantly, this embedding approximation overcomes the DFT limitations in describing charge transfer, multiconfigurational character, and excited states of adsorbed molecules and/or of point defects by means of explicitly correlated wave functions.^56^ These embedded correlated wave function approaches can be applied to the study of photocatalytic nanoparticles,^57,58^ or to extended systems, like transition metal oxide semiconductors, providing a straightforward path for the inclusion of dopants.^59^

The methods described so far provide a static representation of the systems as illustrated in Figure 4a,b, thus ignoring the time domain of the different stages of the global photocatalytic process. Clearly, to acquire a thorough understanding of such nonequilibrium phenomena, dynamic simulations are required (*cf*. Figure 4c). However, most of the theoretical tools available to model excited-state dynamics are intended to study molecular/small systems, since the proper description of the excited states involves advanced electronic structure techniques. Due to the high computational demands, these methods are hard to apply to extended systems. As an alternative, Akimov and Prezhdo^60,61^ have developed a new nonadiabatic molecular dynamics (NAMD) approach that can be applied to the study of condensed matter systems. The developed PYXAID (PYthon eXtension for Ab Initio Dynamics) program bases the reduction of computational cost on the implementation of the neglect of back-reaction approximation (NBRA), which assumes that the nuclear dynamics is not strongly affected by the electronic dynamics, which can be a reasonable assumption for several processes taking place in condensed matter systems, but fails to catch significant nuclear structural reorganizations, such as isomerization, fragmentation, bond breaking, or bond formation processes. The NAMD calculations are combined with electronic structure calculations based on real-time TD-DFT, and the PYXAID program can be interfaced to several commercial and open-access periodic calculation packages. However, the calculation of the adiabatic electronic states is limited to the DFT+*U* approach, not being able to use more accurate electronic structure methodologies like hybrid DFT or *GW* methods mentioned earlier.

The NAMD methodology has been applied to study the mechanism and the time scales of different stages of the photocatalytic process in nanoparticles, surfaces, and bulk materials. This has allowed investigating the time evolution of processes of interest in photocatalysis at the atomic level so as to reach a time-resolved description of charge carrier dynamics, excitation energy transfer, vibrational excitation energy relaxation, or spin dynamics and electronic vibrational decoherence, to name a few. NAMD also provides atomistic insight into the origin of quasiparticles, excitons, or polarons. One critical aspect of this approach is the high computational burden needed to reach simulations in the picosecond scale. This is opposite ground-state AIMD simulation based on DFT where simulations of tens of picoseconds are easily attainable. Despite these limitations, NAMD can be used to guide the interpretation of experiments and to design new materials.

With regard to TiO_2_, the electron–hole recombination dynamics in TiO_2_ nanoparticles of different size and shape have been studied, showing that the charge recombination time becomes larger when increasing the nanoparticle size.^62^ The dynamics of photoinduced polarons and electron–hole recombination in bulk TiO_2_ rutile have been analyzed at different temperatures, revealing that at low temperature polarons are formed in the femtosecond scale and localized on single Ti atoms while electron–hole recombination occurs in nanoseconds; in contrast, at high temperatures the polaron delocalizes, inducing electron–hole recombination in picoseconds, thus quenching the polaron formation.^63^ The dynamics of photoprocesses at surfaces has also been investigated, *e.g*., the time scales of photoinduced electron transfer and electron–phonon energy relaxation processes in the graphene–TiO_2_ rutile interface^64^ or the nonradiative charge trapping and recombination dynamics in Cu-doped anatase TiO_2_(101) surface, showing that the presence of an adsorbed H atom on the single-atom catalyst enhances the activity of photoelectrochemical water splitting.^65^ Another key aspect, not described in this Perspective, is the detailed reactivity in the excited state. The interaction of the reactants, intermediates, and products in the excited state differs, in principle, from that corresponding to the electronic ground state, a field that is to be explored. In this context, it may appear that the interaction between reactants and the photoactive material when activated becomes too weak or too strong, thus limiting the photocatalytic process. Thermodynamic and kinetic aspects in the excited states will be necessary to successfully complete the new road map toward photocatalysis in the excited state.

In conclusion, we have discussed the state-of-the-art in computational modeling approaches to photocatalysis with particular emphasis on the strategies that aim to go beyond the static DFT picture, which provides rather limited information on the properties of the relevant excited states. From the overall discussion, it is suggested that the way to realize an accurate and physically meaningful description of heterogeneously photocatalyzed processes should include (*i*) localizing the regions in the photoactive material where the electrons and holes are placed, which requires static calculations only; (*ii*) determining the lifetime of the photogenerated species, as in NAMD; and (*iii*) moving toward strategies able to explore the reaction mechanisms on the relevant excited-state potential energy surface(s). Clearly, new developments and improving the existing ones are needed, but achieving the goal of shedding light on chemical processes heterogeneously photocatalyzed is worth the effort.
